# Supplementary material for: Van der Waals isotope heterostructures for engineering phonon polariton dispersions
Source: Nat Commun. 2023 Aug 8;14:4782. doi: 10.1038/s41467-023-40449-w (PMC10409777; doi:10.1038/s41467-023-40449-w)
Supplement: Supplementary file 1 — Supplementary Information [file 41467_2023_40449_MOESM1_ESM.pdf]

Supplementary Information for
Van der Waals isotope heterostructures for engineering phonon polariton dispersions

M. Chen[1†], Y. Zhong[2†], E. Harris[3], J. Li[4], Z. Zheng[5], H. Chen[2, 6], J.-S. Wu[7], P. Jarillo-Herrero[5], Q. Ma[3], J. H. Edgar[4], X. Lin[2], S. Dai[1]*

[1]*Materials Research and Education Center, Department of Mechanical Engineering, Auburn University, Auburn, Alabama 36849, USA*

[2]*Interdisciplinary Center for Quantum Information, State Key Laboratory of Modern Optical Instrumentation, ZJU-Hangzhou Global Science and Technology Innovation Center, Zhejiang University, Hangzhou 310027, China*

[3]*Department of Physics, Boston College, Chestnut Hill, Massachusetts 02467, USA*

[4]*Tim Taylor Department of Chemical Engineering, Kansas State University, Manhattan, Kansas 66506, USA*

[5]*Department of Physics, Massachusetts Institute of Technology, Cambridge, Massachusetts 02139, USA*

[6]*International Joint Innovation Center, The Electromagnetics Academy at Zhejiang University, Zhejiang University, Haining 314400, China*

[7]*Department of Photonics and Institute of Electro-Optical Engineering, National Yang Ming Chiao Tung University, Hsinchu 30050, Taiwan*

[†]These authors contribute equally

*Correspondence to: sdai@auburn.edu

Table of Contents

I. Supplementary Figures

Figure 1……………………………………………………………………………3
Figure 2……………………………………………………………………………4
Figure 3……………………………………………………………………………5
Figure 4……………………………………………………………………………6
Figure 5……………………………………………………………………………7
Figure 6……………………………………………………………………………9
Figure 7…………………………………………………………………………..10
Figure 8…………………………………………………………………………..11
II. Supplementary Notes
Note 1: Fourier Transform (FT) analysis of polaritons in isotopic heterostructures…….13
Note 2: Calculation of ω-k dispersions of HPPs in isotopic heterostructures…………...13
Note 3: Supplementary ω-k dispersions reveal the engineering of HPPs in isotopic heterostructures by varying the thicknesses of ^{10}B and ^{11}B building blocks……13
Note 4: Geometrical optics interpretation for HPPs in isotopic heterostructures………..13
4.1. HPPs in a monoisotopic ^{11}B or ^{10}B hBN slab………………………………14
4.2. HPPs in 2-slab isotopic heterostructures……………………………………14
4.3. HPPs in 3-slab isotopic heterostructures……………………………………15
Note 5: Electromagnetic interaction leads to various HPP dispersions in isotopic heterostructures……………………………………….…………………………16
5.1. Engineering HPP dispersions in thickness-symmetric ^{11}B|^{10}B|^{11}B isotopic heterostructures by varying the ^{10}B slab thickness…………………………16
5.2. Engineering HPP dispersions in thickness-asymmetric ^{11}B|^{10}B|^{11}B isotopic heterostructures by varying the ^{10}B slab thickness…………………………17
III. Supplementary Notes…………………………………………………………………18

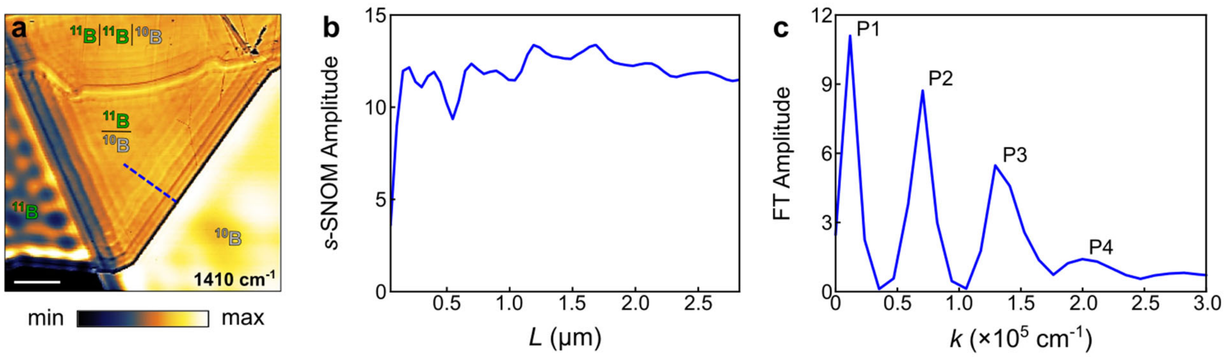

Supplementary Figure 1| Example Fourier Transform (FT) analysis of hyperbolic phonon polaritons (HPPs) in ^{10}B-^{11}B isotopic heterostructures. **a**, s-SNOM amplitude image of ^{10}B-^{11}B isotopic heterostructure at IR frequency ω = 1410 cm^{-1}. Scale bar: 1 μm. **b**, Line profile cut from (**a**) along the blue dashed line. *L* denotes the distance from the ^{11}B|^{10}B heterostructure edge. **c**, FT spectrum of the line profile in (**b**). P1, P2, P3, and P4 denote the resonances of considerable intensity.

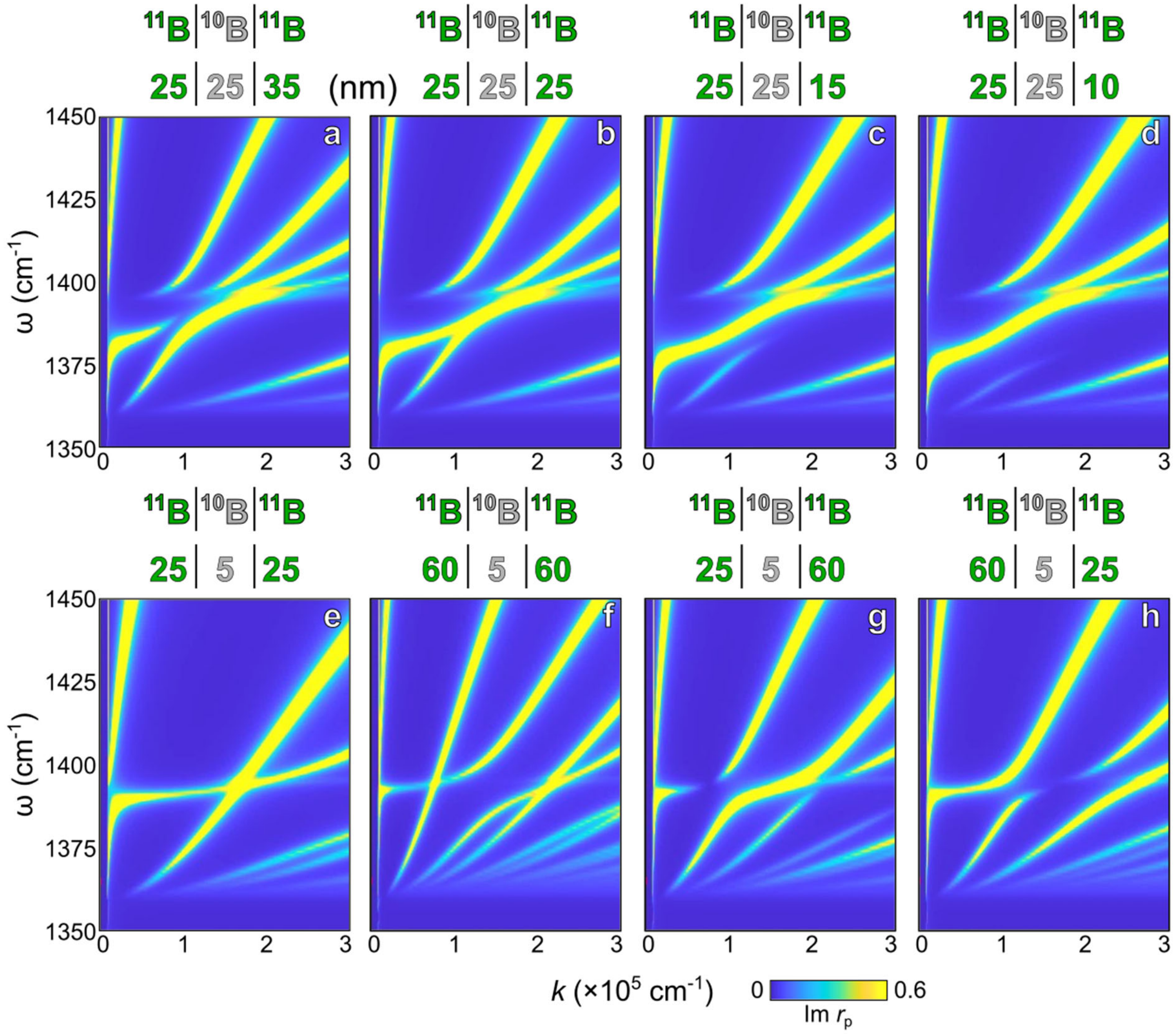

Supplementary Figure 2 | Supplementary ω-*k* dispersions reveal the tailoring of HPPs in isotopic heterostructures by varying the thicknesses of the ^{10}B and ^{11}B building blocks. **a**–**h**, HPPs in 3-slab isotopic heterostructures with the identical composition and stacking ^{11}B|^{10}B|^{11}B but with the thickness 25|25|35 nm (**a**), 25|25|25 nm (**b**), 25|25|15 nm (**c**), 25|25|10 nm (**d**), 25|5|25 nm (**e**), 60|5|60 nm (**f**), 25|5|60 nm (**g**), and 60|5|25 nm (**h**).

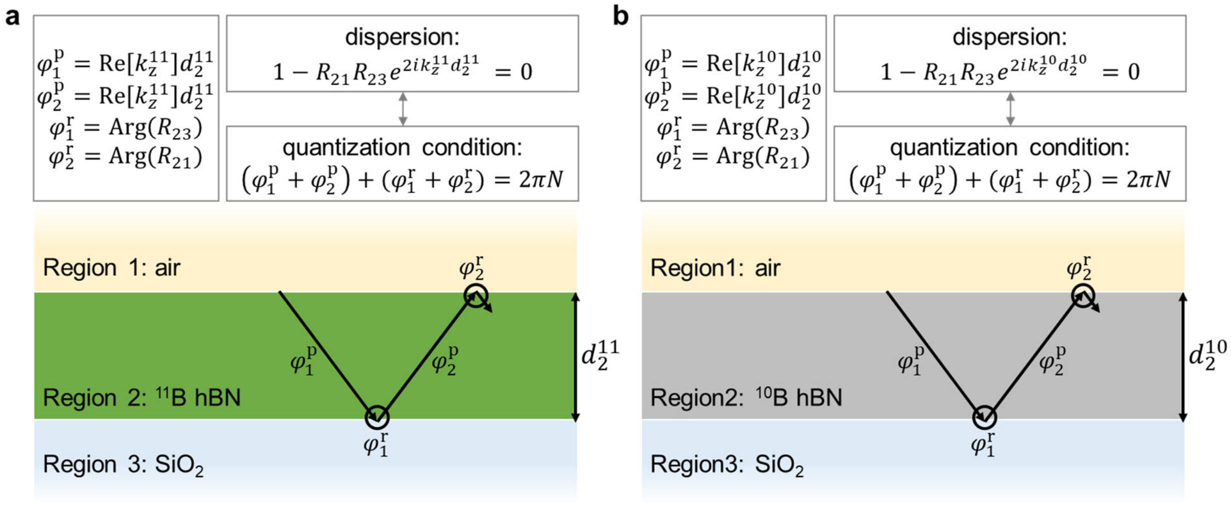

Supplementary Figure 3 | Geometric optics model for hyperbolic phonon polaritons in ^{11}B (a) and ^{10}B (b) hBN. The monoisotopic ^{11}B(^{10}B) hBN with a thickness of $d_2^{11}(d_2^{10})$ is placed on the SiO_2 substrate. The arrows denote the propagation path of HPPs. In one "down-and-up" period, the total phase accumulation $\Delta\varphi$ consists of the propagation phase $\varphi_1^{\mathrm{p}} + \varphi_2^{\mathrm{p}}$ in ^{11}B(^{10}B) hBN, and the reflection phase $\varphi_{1(2)}^{\mathrm{r}}$ at the interface of hBN-SiO_2(air). According to the quantization condition, the total phase accumulation is equal to $2\pi N$, where N is an integer.

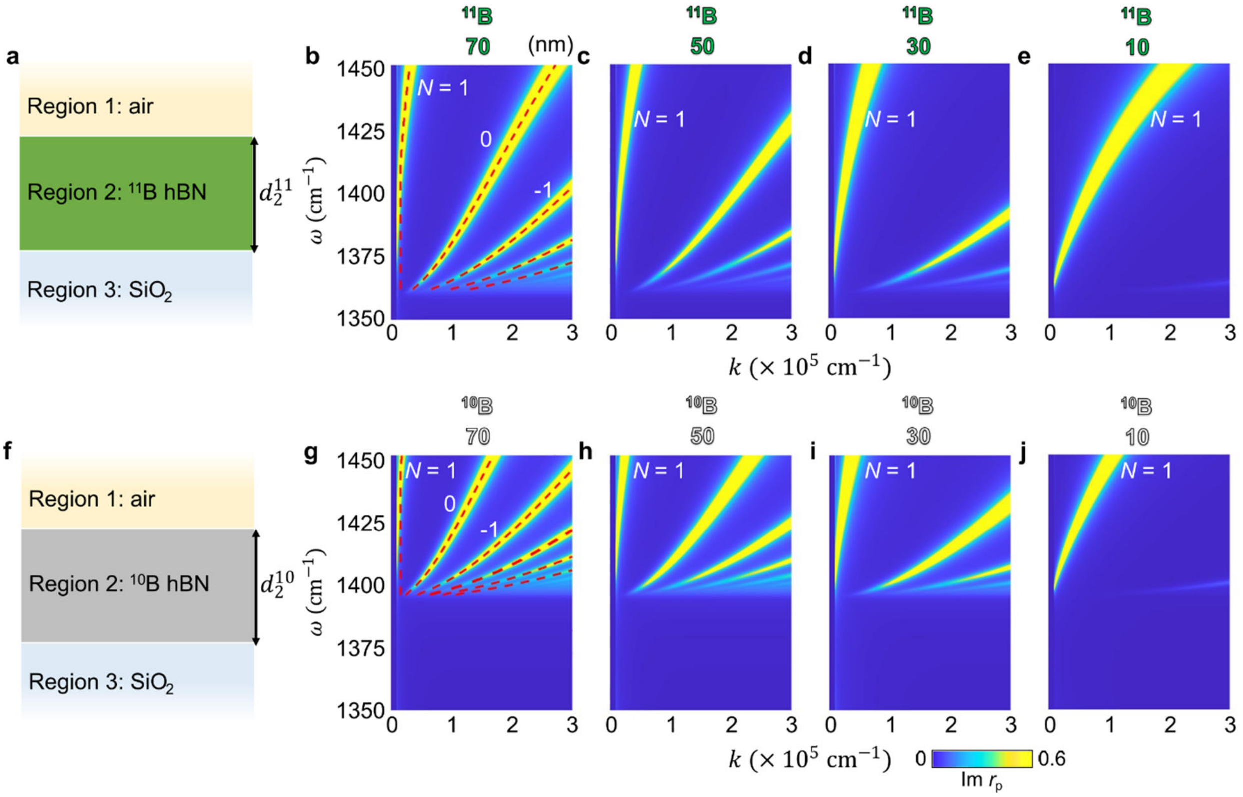

Supplementary Figure 4 | ω-k dispersions in ^{11}B(^{10}B) hBN with various thicknesses. (a) Geometrical optics model for HPPs in ^{11}B hBN. In one "down-and-up" period, $\Delta\varphi = 2\pi N$. Specifically, $N = 1$ for the first HPP branch and N decreases by 1 for the subsequent HPPs branches. **(b–e)** HPP dispersions in ^{11}B hBN with a thickness of 70 nm (b), 50 nm (c), 30 nm (d), and 10 nm (e). **(f)** Geometrical optics model for HPPs in ^{10}B hBN. **(g–j)** The HPP dispersions in ^{10}B hBN with the thickness of 70 nm (g), 50 nm (h), 30 nm (i), and 10 nm (j). Similarly, the HPP branches shift towards large k at the decreasing slab thickness. HPP dispersions from the geometrical optics model (e.g., red dashed curves in panels b and g) agree excellently with the calculations of Im r_p (false color, Supplementary Note 2).

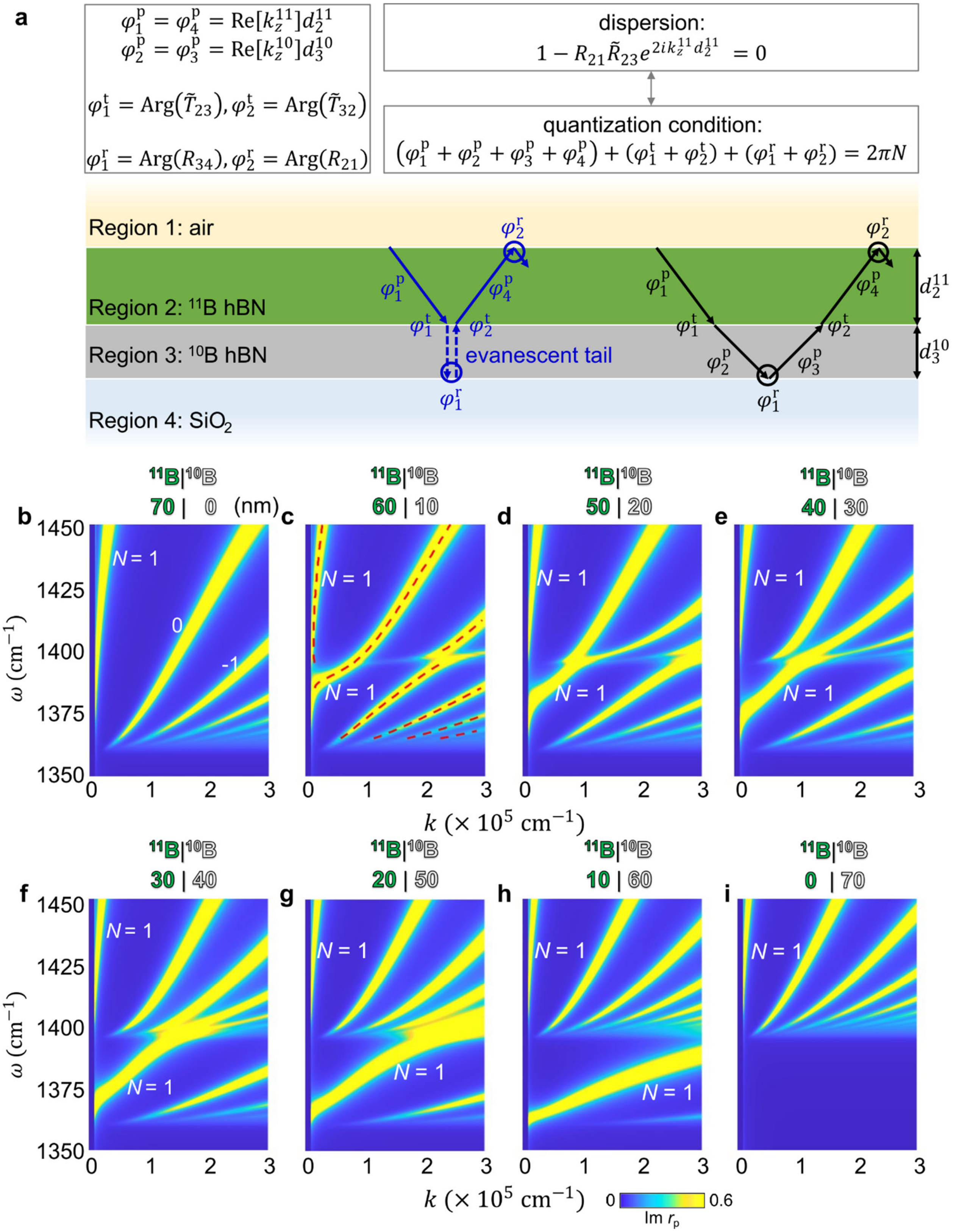

Supplementary Figure 5 | HPPs in 2-slab isotopic heterostructures. (a) Geometrical optics model for HPPs in 2-slab isotopic heterostructure with the stacking of ^{11}B|^{10}B. The heterostructure

is placed on the SiO_2 substrate, and the thicknesses of ^{11}B and ^{10}B hBN are denoted by d_2^{11} and d_3^{10}, respectively. The blue arrows denote the propagation path of HPPs at ω < 1395 cm^{-1}. The black arrows denote the propagation path of HPPs at ω > 1395 cm^{-1}. In one "down-and-up" period, $\Delta\varphi$ consists of the propagation phases $\varphi_1^{\mathrm{p}} + \varphi_4^{\mathrm{p}} (\varphi_2^{\mathrm{p}} + \varphi_3^{\mathrm{p}})$ inside the $^{11}B(^{10}B)$ slab, the transmission phases $\varphi_{1,2}^{\mathrm{t}}$ at the inner interface of ^{11}B-^{10}B, and the reflection phases $\varphi_{1(2)}^{\mathrm{r}}$ at the interface of ^{10}B hBN-SiO_2(^{11}B hBN-air). At ω < 1395 cm^{-1}, the propagation phase $\varphi_2^{\mathrm{p}} + \varphi_3^{\mathrm{p}} = 2\mathrm{Re}[k_z^{10}]d_3^{10}$ in ^{10}B slab is zero if the material loss is neglected, while the transmission phases $\varphi_{1,2}^{\mathrm{t}}$ at the inner interface of ^{11}B-^{10}B are non-neglectable and cause phase jumping. **(b-i)** The dispersions for HPPs in 2-slab isotopic heterostructures with a total thickness of 70 nm (i.e., $d_2^{11} + d_3^{10} = 70$ nm). The red dashed curves are results from the geometrical optics model, while the false color indicates the Im r_{p} calculations.

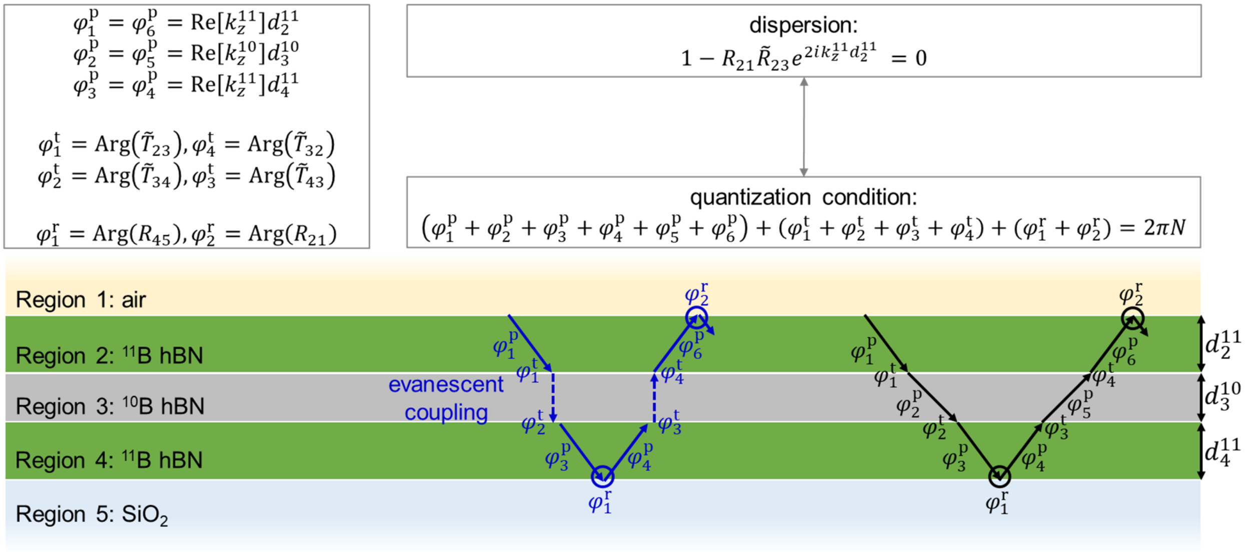

Supplementary Figure 6 | Geometrical optics model for HPPs in 3-slab isotopic heterostructures with a representative stacking of ^{11}B|^{10}B|^{11}B. The heterostructure is placed on the SiO_2 substrate. The blue arrows denote the propagation path of HPPs at $\omega < 1395$ cm^{-1}. The black arrows represent the propagation path of HPPs at $\omega > 1395$ cm^{-1}. The propagation phases consist of $\varphi_1^{\mathrm{p}} + \varphi_6^{\mathrm{p}}$ in the top ^{11}B slab, $\varphi_2^{\mathrm{p}} + \varphi_5^{\mathrm{p}}$ in the ^{10}B slab and $\varphi_3^{\mathrm{p}} + \varphi_4^{\mathrm{p}}$ in the bottom ^{11}B slab. The transmission phase consists of $\varphi_{1,4}^{\mathrm{t}}$ at the first ^{11}B-^{10}B inner interface and $\varphi_{2,3}^{\mathrm{t}}$ at the second ^{10}B-^{11}B inner interface. The reflection phase consists of φ_1^{r} at the interface of ^{10}B hBN-SiO_2 and φ_2^{r} at the interface of ^{11}B hBN-air. The total phase accumulation $\Delta\varphi$ includes all the phases mentioned above and should be $2\pi N$ to satisfy the quantization condition.

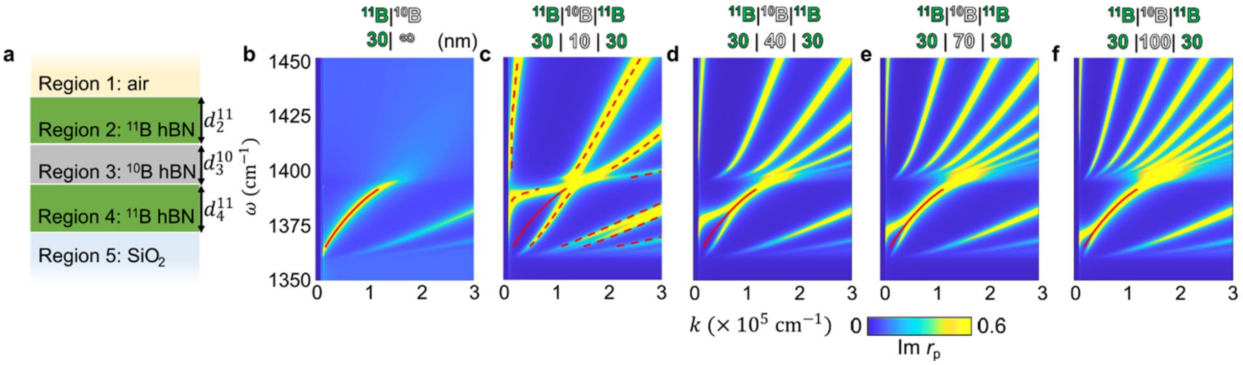

Supplementary Figure 7 | HPP dispersions in thickness-symmetric 3-slab isotopic heterostructures with the stacking of ^{11}B|^{10}B|^{11}B. (a) Schematic for the 3-slab isotopic heterostructure with the stacking of ^{11}B|^{10}B|^{11}B. **(b)** HPP dispersion in the ^{11}B slab (thickness 30 nm) on infinitely-thick ^{10}B hBN. The solid red curve tracks the first (from the left of the *k* axis) HPP branch. **(c-f)** HPP dispersions 3-slab isotopic heterostructures. The thicknesses of the first (d_2^{11}) and second (d_4^{11}) ^{11}B slab are the same (e.g., $d_2^{11} = d_4^{11} = 30$ nm), and the thickness of the middle ^{10}B slab (d_3^{10}) varies from 10 (c), 40 (d), 70 (e), to 100 (f) nm. The red dashed curves are results from the geometrical optics model, while the false color indicates the Im r_p calculations.

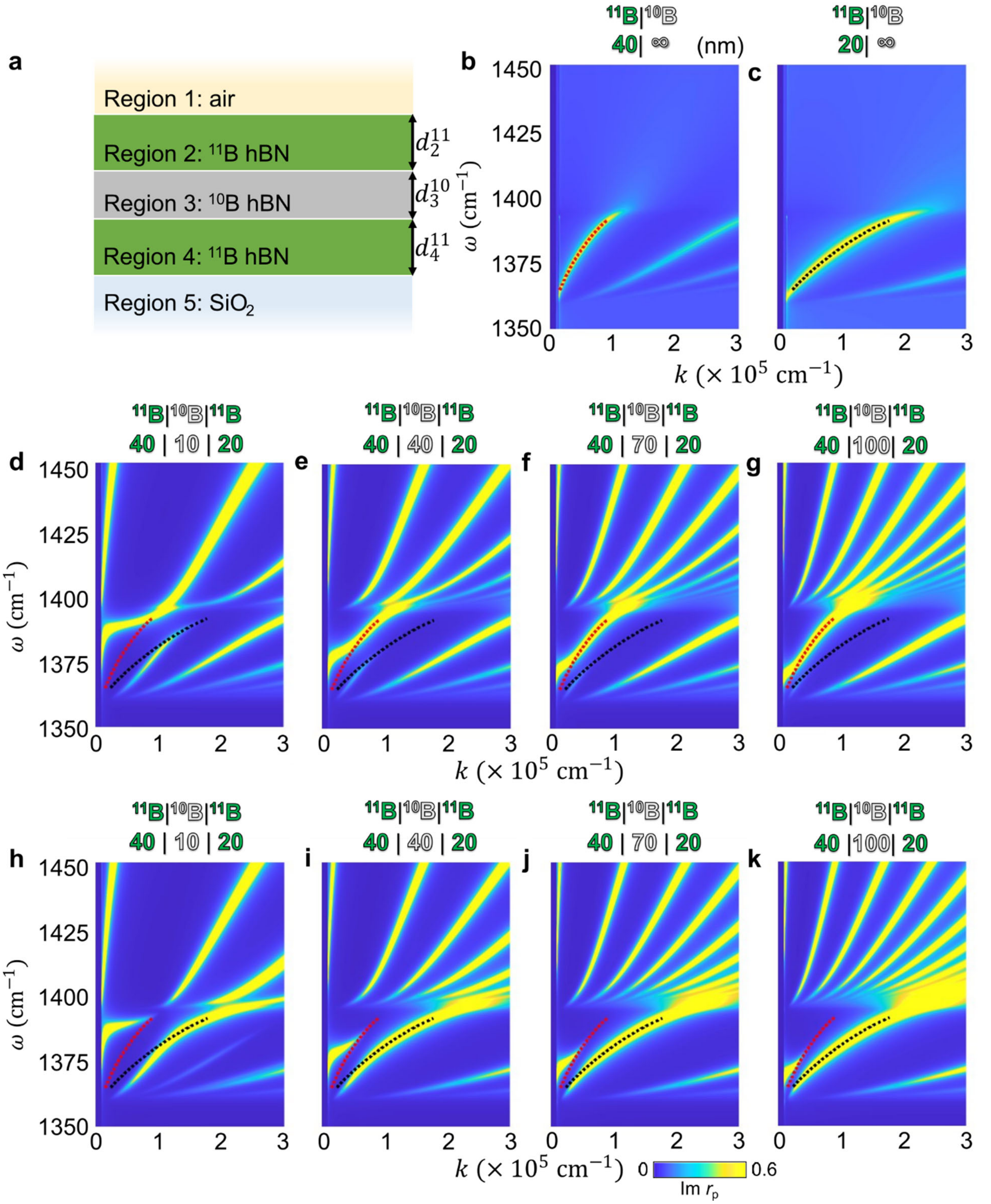

Supplementary Figure 8 | HPP dispersions in thickness-asymmetric 3-slab isotopic heterostructures with the stacking of ^{11}B|^{10}B|^{11}B. (a) Schematic for the 3-slab isotopic heterostructure with the stacking of ^{11}B|^{10}B|^{11}B. **(b)** HPP dispersion in ^{11}B slab (thickness 40 nm) on infinitely-thick ^{10}B hBN. The red dotted line tracks the first (from the left) HPP branch. **(c)** HPP dispersion in ^{11}B slab (thickness 20 nm) on infinitely-thick ^{10}B hBN. The black dotted line tracks

the first (from the left) HPP branch. **(d-g)** HPP dispersions thickness-asymmetric 3-slab isotopic heterostructures. The thicknesses of the top (d_2^{11}) and bottom (d_4^{11}) ^{11}B slabs are 20 and 40 nm, respectively. And the thickness of the middle ^{10}B slab (d_3^{10}) varies from 10 (c), 40 (d), 70 (e), to 100 (f) nm. **(h-k)** Dispersions for HPPs in the asymmetric 3-slab isotopic heterostructure where the thicknesses of the top (d_2^{11}) and bottom (d_4^{11}) ^{11}B slabs are 20 and 40 nm, respectively. And the thickness of the middle ^{10}B slab (d_3^{10}) varies from 10 (h), 40 (i), 70 (j), to 100 (k) nm.

Supplementary Note 1. Fourier Transform (FT) analysis of polaritons in isotopic heterostructures

The experimental data (red circles in Fig. 3–4, main text) on energy-momentum (ω-*k*) dispersion of hyperbolic phonon polaritons (HPPs) were obtained by Fourier Transfer (FT) analysis of the scattering-type scanning near-field optical microscopy (s-SNOM) images (e.g., Fig. 2 in the main text). In Supplementary Fig. 1, we provide an example FT analysis of HPPs in $^{11}B|^{10}B$ isotopic heterostructure. At a representative frequency ω = 1410 cm^{-1}, the line profile (Supplementary Fig. 1b) of s-SNOM amplitude was extracted from the cut of the s-SNOM image (blue dashed line in Supplementary Fig. 1a). FT of the s-SNOM line profile reveals a series of resonances (of considerable amplitude) P1, P2, P3, and P4 at 0.1, 0.7, 1.3, and 2.0 × 10^5 cm^{-1}. Each resonance reveals a single-period oscillation of the s-SNOM amplitude representing the standing waves of HPPs. The polariton momentum *k* can be extracted from the resonance peak position in the FT spectrum (Supplementary Fig. 1c). Since P3 of 1.3 × 10^5 cm^{-1} is about twice P2 of 0.7 × 10^5 cm^{-1}. P2 and P3 are treated as tip-launched and edge-launched HPPs[1] from the same dispersion branch. Therefore, the extracted *k* for HPPs at ω = 1410 cm^{-1} in the $^{11}B|^{10}B$ isotopic heterostructure are: 0.1, 1.3, and 2.0 × 10^5 cm^{-1} (denoted as red circles in Fig. 3a in the main text).

Supplementary Note 2. Calculation of ω-*k* dispersions of HPPs in isotopic heterostructures

The energy-momentum (ω-*k*) dispersions of HPPs in ^{10}B-^{11}B isotopic heterostructures can be modeled by calculating the complex reflectivity r_p[2]. In the calculation, thicknesses, and permittivity of the ^{10}B and ^{11}B hBN building blocks are input from atomic force microscopy (AFM) data and the literature[3]. The false color in Fig. 3–4 in the main text and Supplementary Fig. 2 represents the imaginary part of the calculated complex reflectivity Im r_p.

Supplementary Note 3. Supplementary ω-*k* dispersions reveal the engineering of HPPs in isotopic heterostructures by varying the thicknesses of ^{10}B and ^{11}B building blocks

In addition to the combined s-SNOM data and electromagnetics (EM) calculation in Fig. 4 in the main text, here we provide a systematic investigation of engineering HPPs in isotopic heterostructures by varying the thicknesses of the ^{10}B and ^{11}B building blocks. We showcase the degree of freedom of building block thicknesses in 3-slab isotopic heterostructures—with identical composition and stacking $^{11}B|^{10}B|^{11}B$ yet different thicknesses (denoted under the stacking) of the ^{10}B and ^{11}B slab—with a variety of ω-*k* dispersions (Supplementary Fig. 2a-h). By varying the thickness of the bottom ^{11}B slab (Supplementary Fig. 2a-d), the dispersions exhibit approaching branches at the thickness of 25|25|35 nm (Supplementary Fig. 2a), merging branches at the thickness of 25|25|25 nm (Supplementary Fig. 2b), and unbalanced branches at the thickness of 25|25|15 nm and 25|25|10 nm (Supplementary Fig. 2c-d). Varying the thicknesses of top ^{11}B and bottom ^{11}B with the identical middle ^{10}B slab also affect the polariton dispersions, including a series of connecting (Supplementary Fig. 2e-f) and disconnecting (Supplementary Fig. 2g-h) branches.

Supplementary Note 4. Geometrical optics interpretation for HPPs in isotopic heterostructures

In the main text, isotope van der Waals (vdW) heterostructuring is demonstrated to engineer HPPs into a variety of ω-k dispersions by varying the composition, stacking, and thickness of monoisotopic ^{10}B and ^{11}B vdW building blocks. Here we describe the geometrical optics model to interpret these engineered HPPs.

Supplementary Note 4.1. HPPs in a monoisotopic ^{11}B or ^{10}B hBN slab

We begin with the case of HPPs in the monoisotopic ^{11}B or ^{10}B hBN (referred to as ^{11}B or ^{10}B slab) on a dielectric substrate (e.g., SiO_2). The different atomic masses of B yield the Reststrahlen band at ω = 1359.8–1608.7 cm^{-1} and ω = 1394.5–1650 cm^{-1} for ^{11}B and ^{10}B hBN, respectively. Within the Reststrahlen band, HPPs are mainly confined inside the hBN slab, and they propagate via total internal reflections at the interface with air and SiO_2 (see the paths denoted by black arrows in Supplementary Fig. 3). Per the Fabry-Perot quantization condition[2, 4], the total phase accumulation $\Delta\varphi$ of HPPs propagating "down-and-up" should be:

$$\Delta\varphi = 2\mathrm{Re}[k_z^{11}]d_2^{11} + \mathrm{Arg}(R_{21}) + \mathrm{Arg}(R_{23}) = 2\pi N \quad (1)$$

where N is an integer. $\varphi_1^{\mathrm{p}} = \varphi_2^{\mathrm{p}} = \mathrm{Re}\left[k_z^{11(10)}\right]d_2^{11(10)}$ are the propagation phases inside the ^{11}B(^{10}B) slab, where $k_z^{11(10)}$ from $\frac{\left(k_z^{11(10)}\right)^2}{\varepsilon_t^{11(10)}} + \frac{\left(k^{11(10)}\right)^2}{\varepsilon_z^{11(10)}} = (\frac{\omega}{c})^2$ is the vertical momentum of HPPs and $d_2^{11(10)}$ is the thickness of the ^{11}B(^{10}B) slab. $\varphi_1^{\mathrm{r}} = \mathrm{Arg}(R_{23})$ and $\varphi_2^{\mathrm{r}} = \mathrm{Arg}(R_{21})$ are the reflection phases at the interface of hBN-SiO_2 and hBN-air, respectively. $\mathrm{Arg}(R_{23}) = \mathrm{atan}\,(\frac{\mathrm{Im}[R_{23}]}{\mathrm{Re}[R_{23}]})$ and $\mathrm{Arg}(R_{21}) = \mathrm{atan}\,(\frac{\mathrm{Im}[R_{21}]}{\mathrm{Re}[R_{21}]})$, where $R_{23} = \frac{k_z^2/\varepsilon_2 - k_z^3/\varepsilon_3}{k_z^2/\varepsilon_2 + k_z^3/\varepsilon_3}$ and $R_{21} = \frac{k_z^2/\varepsilon_2 - k_z^1/\varepsilon_1}{k_z^2/\varepsilon_2 + k_z^1/\varepsilon_1}$ are the complex reflectivities. Here, ε_{1-3} is the permittivity of Region 1–3 and k_z^{1-3} is the vertical momentum of HPPs in Region 1-3. Specifically, we have $\varepsilon_2 = \varepsilon_t^{11(10)}$, $k_z^2 = k_z^{11(10)}$.

Equation (1) indicates that HPPs at different branches (different N) share the same propagation path but possess different $\Delta\varphi$. Specifically, $\Delta\varphi$ for adjacent HPP branches differ by 2π (Supplementary Fig. 4b-e, g-j). In the calculation, we regulate $\varphi_{1(2)}^{\mathrm{r}} \in [0,2\pi)$. Therefore, $N = 1$ for the first (from left in the k axis) HPP branch, and N decreases for the subsequent HPP branches. Equation (1) also reveals the thickness dependence of HPPs. At the same dispersion branch (fixed N), if $d_2^{11(10)}$ decreases, $k_z^{11(10)}$ and k (see Equation (1) in the main text) have to increase. As a result, HPP branches will shift to larger k.

The geometrical optics model produces HPP dispersions (dashed curves in Supplementary Fig. 4, Fig. 5, Fig. 7) in accord with the reflectivity calculation in Supplementary Note 2 (false color).

Supplementary Note 4.2. HPPs in 2-slab isotopic heterostructures

In this subsection, we discuss the geometrical optics model for HPPs in 2-slab isotopic heterostructures with the stacking of ^{11}B|^{10}B.

At ω > 1395 cm^{-1}, HPPs propagate as diffractionless rays in both ^{11}B and ^{10}B slabs, and the propagation path is represented by the black arrows in Supplementary Fig. 5a. The quantization condition yields

$$\Delta\varphi = 2\mathrm{Re}[k_z^{11}]d_2^{11} + 2\mathrm{Re}[k_z^{10}]d_3^{10} + \mathrm{Arg}(\tilde{T}_{23}) + \mathrm{Arg}(\tilde{T}_{32}) + \mathrm{Arg}(R_{21}) + \mathrm{Arg}(R_{34}) = 2\pi N. \quad (2)$$

Here, $\varphi_1^{\mathrm{p}} = \varphi_4^{\mathrm{p}} = \mathrm{Re}[k_z^{11}]d_2^{11}$ are the propagation phases inside the ^{11}B slab, where d_2^{11} is the thickness. $\varphi_2^{\mathrm{p}} = \varphi_3^{\mathrm{p}} = \mathrm{Re}[k_z^{10}]d_3^{10}$ are the propagation phases inside the ^{10}B slab and d_3^{10} is the thickness. $\varphi_1^{\mathrm{r}} = \mathrm{Arg}(R_{34})$ and $\varphi_2^{\mathrm{r}} = \mathrm{Arg}(R_{21})$ are the reflection phase shifts at the interface of ^{10}B hBN-SiO_2 and ^{11}B hBN-air, respectively. $\varphi_1^{\mathrm{t}} = \mathrm{Arg}(\tilde{T}_{23})$ and $\varphi_2^{\mathrm{t}} = \mathrm{Arg}(\tilde{T}_{32})$ are the transmission phases at the inner interface of ^{11}B-^{10}B inside the isotopic heterostructure. In the calculation, we regulate $\mathrm{Arg}(R_{21}) = \mathrm{atan}\,(\frac{\mathrm{Im}[R_{21}]}{\mathrm{Re}[R_{21}]})$ and $\mathrm{Arg}(R_{34}) = \mathrm{atan}\,(\frac{\mathrm{Im}[R_{34}]}{\mathrm{Re}[R_{34}]})$ in $[0,2\pi)$, and $\mathrm{Arg}(\tilde{T}_{23}) = \mathrm{atan}\,(\frac{\mathrm{Im}[\tilde{T}_{23}]}{\mathrm{Re}[\tilde{T}_{23}]})$ and $\mathrm{Arg}(\tilde{T}_{32}) = \mathrm{atan}\,(\frac{\mathrm{Im}[\tilde{T}_{32}]}{\mathrm{Re}[\tilde{T}_{32}]})$ in $[-\pi,\pi)$. $R_{21} = \frac{k_z^2/\varepsilon_2 - k_z^1/\varepsilon_1}{k_z^2/\varepsilon_2 + k_z^1/\varepsilon_1}$ and $R_{34} = \frac{k_z^3/\varepsilon_3 - k_z^4/\varepsilon_4}{k_z^3/\varepsilon_3 + k_z^4/\varepsilon_4}$ are the complex reflectivities, and specifically we have $\varepsilon_2 = \varepsilon_t^{11}$, $\varepsilon_3 = \varepsilon_t^{10}$, $k_z^2 = k_z^{11}$, $k_z^3 = k_z^{10}$. $\tilde{T}_{23}$ and $\tilde{T}_{32}$ are the generalized transmittances[4, 5]:

$$\tilde{T}_{23} = \frac{T_{23}}{1 + R_{23}R_{34}e^{2ik_z^3 d_3^{10}}} \tag{3}$$

$$\tilde{T}_{32} = \frac{T_{32}}{1 + R_{32}R_{21}e^{2ik_z^2 d_2^{11}}} \tag{4}$$

where $R_{23} = \frac{k_z^2/\varepsilon_2 - k_z^3/\varepsilon_3}{k_z^2/\varepsilon_2 + k_z^3/\varepsilon_3}$, $R_{32} = \frac{k_z^3/\varepsilon_3 - k_z^2/\varepsilon_2}{k_z^3/\varepsilon_3 + k_z^2/\varepsilon_2}$, $T_{23} = \frac{2k_z^2/\varepsilon_2}{k_z^2/\varepsilon_2 + k_z^3/\varepsilon_3}$, $T_{32} = \frac{2k_z^3/\varepsilon_3}{k_z^3/\varepsilon_3 + k_z^2/\varepsilon_2}$. $k_z^{11} \neq k_z^{10}$, therefore the speed of HPP phase accumulation varies in ^{11}B and ^{10}B subregions inside the isotopic heterostructure. For example, at the identical total thickness ($d_2^{11} + d_3^{10} = 70$ nm), the HPP dispersions vary evidently with d_2^{11} and d_3^{10} (Supplementary Fig. 5b-i).

At $\omega < 1395$ cm^{-1}, HPPs propagate as diffractionless rays in ^{11}B hBN but evanescently decay in ^{10}B hBN; see the blue arrows in Supplementary Fig. 5a. For conceptual brevity, the material loss is neglected. Thus, $\varphi_2^{\mathrm{p}} = \varphi_3^{\mathrm{p}} = \mathrm{Re}[k_z^{10}]d_3^{10} = 0$, indicating that even if electromagnetic fields of the HPPs can enter ^{10}B hBN, there will be no phase accumulation therein. However, the inner interface of ^{11}B-^{10}B inside the 2-slab isotopic heterostructure will introduce a non-negligible phase jumping $\varphi_1^{\mathrm{t}} + \varphi_2^{\mathrm{t}}$, therefore allowing the engineering of HPP dispersions. In addition, the HPP branches split around $\omega \approx 1395\ \mathrm{cm}^{-1}$, due to the hyperbolicity/ellipticity to hyperbolicity/hyperbolicity transformation of the isotopic heterostructure and the phase jumping at the ^{11}B-^{10}B inner interface.

Supplementary Note 4.3. HPPs in 3-slab isotopic heterostructures

In this subsection, we analyze HPPs in a representative 3-slab isotopic heterostructure with the stacking of ^{11}B|^{10}B|^{11}B.

At $\omega > 1395$ cm^{-1}, HPPs propagate as diffractionless rays in ^{11}B and ^{10}B slabs (see black arrows in Supplementary Fig. 6). The total phase accumulation $\Delta\varphi$ in one "down-and-up" period consists of the propagation phases $\varphi_{1-6}^{\mathrm{p}}$, the transmission phases $\varphi_{1-4}^{\mathrm{t}}$, and the reflection phases $\varphi_{1,2}^{\mathrm{r}}$.

$$\Delta\varphi = 2\mathrm{Re}[k_z^{11}](d_2^{11} + d_4^{11}) + 2\mathrm{Re}[k_z^{10}]d_3^{10} + \mathrm{Arg}(\tilde{T}_{23}) + \mathrm{Arg}(\tilde{T}_{32}) + \mathrm{Arg}(\tilde{T}_{34}) + \mathrm{Arg}(\tilde{T}_{43}) + \mathrm{Arg}(R_{21}) + \mathrm{Arg}(R_{45}) = 2\pi N. \tag{5}$$

Here, $\varphi_1^{\mathrm{p}} = \varphi_6^{\mathrm{p}} = \mathrm{Re}[k_z^{11}]d_2^{11}$ and $\varphi_3^{\mathrm{p}} = \varphi_4^{\mathrm{p}} = \mathrm{Re}[k_z^{11}]d_4^{11}$ are the propagation phases inside the top and bottom ^{11}B slab, respectively, where d_2^{11} and d_4^{11} are their thicknesses. $\varphi_2^{\mathrm{p}} = \varphi_5^{\mathrm{p}} = \mathrm{Re}[k_z^{10}]d_3^{10}$ are the propagation phases inside the middle ^{10}B slab, where d_3^{10} is the thickness. $\varphi_1^{\mathrm{r}} = \mathrm{Arg}(R_{45})$ and $\varphi_2^{\mathrm{r}} = \mathrm{Arg}(R_{21})$ are the reflection phase at the interface of ^{10}B hBN-SiO_2 and ^{11}B hBN-air, respectively. $\varphi_1^{\mathrm{t}} = \mathrm{Arg}(\tilde{T}_{23})$ and $\varphi_4^{\mathrm{t}} = \mathrm{Arg}(\tilde{T}_{32})$ are the transmission phases

at the first (from top) ^{11}B-^{10}B inner interface. And $\varphi_2^{\mathrm{t}} = \mathrm{Arg}(\tilde{T}_{34})$ and $\varphi_3^{\mathrm{t}} = \mathrm{Arg}(\tilde{T}_{43})$ are the transmission phases at the second (from top) ^{10}B-^{11}B inner interface of hBN. In the calculation, we regulate $\mathrm{Arg}(R_{21}) = \mathrm{atan}\,(\frac{\mathrm{Im}[R_{21}]}{\mathrm{Re}[R_{21}]})$ and $\mathrm{Arg}(R_{45}) = \mathrm{atan}\,(\frac{\mathrm{Im}[R_{45}]}{\mathrm{Re}[R_{45}]})$ in $[0,2\pi)$ and $\mathrm{Arg}(\tilde{T}_{ij}) = \mathrm{atan}\left(\frac{\mathrm{Re}[\tilde{T}_{ij}]}{\mathrm{Im}[\tilde{T}_{ij}]}\right)(i,j = 2,3,4)$ in $[-\pi,\pi)$. $\tilde{T}_{ij}$ are the generalized transmission coefficients:

$$\tilde{T}_{23} = \frac{T_{23}}{1+R_{23}\tilde{R}_{34}e^{2ik_z^3 d_3^{10}}}, \tilde{R}_{34} = \frac{R_{34}+R_{45}e^{2ik_z^4 d_4^{11}}}{1+R_{34}R_{45}e^{2ik_z^4 d_4^{11}}} \quad (6)$$

$$\tilde{T}_{32} = \frac{T_{32}}{1+R_{32}R_{21}e^{2ik_z^2 d_2^{11}}} \quad (7)$$

$$\tilde{T}_{34} = \frac{T_{34}}{1+R_{34}R_{45}e^{2ik_z^4 d_4^{11}}} \quad (8)$$

$$\tilde{T}_{43} = \frac{T_{43}}{1+R_{43}\tilde{R}_{32}e^{2ik_z^3 d_3^{10}}}, \tilde{R}_{32} = \frac{R_{32}+R_{21}e^{2ik_z^2 d_2^{11}}}{1+R_{32}R_{21}e^{2ik_z^2 d_2^{11}}} \quad (9)$$

where $R_{i,i+1} = \frac{k_z^i/\varepsilon_i - k_z^{i+1}/\varepsilon_{i+1}}{k_z^i/\varepsilon_i + k_z^{i+1}/\varepsilon_{i+1}}$, $R_{i,i-1} = -R_{i-1,i}$, $T_{i,i+1} = R_{i,i+1} + 1$, $T_{i,i-1} = R_{i-1,i} + 1$ $(i = 2,3,4)$. Specifically, we have $\varepsilon_2 = \varepsilon_4 = \varepsilon_t^{11}$, $\varepsilon_3 = \varepsilon_t^{10}$, $k_z^2 = k_z^4 = k_z^{11}$, and $k_z^3 = k_z^{10}$.

At ω < 1395 cm^{-1}, HPPs propagate as diffractionless rays in ^{11}B hBN but evanescently decay in ^{10}B hBN. Under this circumstance, $2\mathrm{Re}[k_z^{10}]d_3^{10} = 0$, and the 3-slab isotopic heterostructure can be treated as two ^{11}B slabs separated by a dielectric. The HPPs in the two monoisotopic ^{11}B hBN Electromagnetically interact (see blue arrows in Supplementary Fig. 6). Note that the 3-slab isotopic heterostructure has an additional inner interface compared with the 2-slab isotopic heterostructure. Therefore, there will be additional transmission phases (namely from $(\varphi_1^{\mathrm{t}} + \varphi_2^{\mathrm{t}})$ in 2-slab heterostructure to $(\varphi_1^{\mathrm{t}} + \varphi_4^{\mathrm{t}}) + (\varphi_2^{\mathrm{t}} + \varphi_3^{\mathrm{t}})$ in 3-slab heterostructure). These additional phases result in evident differences for HPP dispersions in 2-slab compared with those in 3-slab heterostructures at ω = 1360–1395 cm^{-1}. Indeed, the ^{11}B-^{10}B inner interfaces inside isotopic heterostructures offer an important degree of freedom to engineer the HPP dispersions.

Supplementary Note 5. Electromagnetic interaction leads to various HPP dispersions in isotopic heterostructures

Supplementary Note 5.1. Engineering HPP dispersions in thickness-symmetric ^{11}B|^{10}B|^{11}B isotopic heterostructures by varying the ^{10}B slab thickness

In this subsection, we exploit Electromagnetic (EM) interactions to analyze the engineered HPP dispersions in a representative isotopic heterostructure ^{11}B|^{10}B|^{11}B by varying the thickness d_3^{10} of the middle ^{10}B slab. We start with the thickness-symmetric heterostructure where the thicknesses of the top and bottom ^{11}B slab are the same (e.g., $d_2^{11} = d_4^{11} = 30$ nm in Supplementary Fig. 8). At ω < 1395 cm^{-1}, HPPs propagate as diffractionless rays in ^{11}B hBN and evanescently decay in ^{10}B hBN. Therefore, the ^{11}B|^{10}B|^{11}B heterostructure can be treated as two ^{11}B slabs separated by a dielectric, where the HPPs in two ^{11}B slabs couple to each other. Here, we analyze in detail the first (from left) two HPPs branches, and the other branches can be interpreted similarly. Without EM interactions, HPP dispersion in the top and bottom ^{11}B slab follows the red solid curves in Supplementary Fig. 7. The EM interactions result in the repulsed[6, 7] HPPs in the ^{11}B|^{10}B|^{11}B heterostructures: the red solid curve splits into two HPP branches (Supplementary Fig. 7c-f, false color). The EM interactions of HPPs in these two ^{11}B slabs depend on their spatial separation (the thickness of the middle ^{10}B slab d_3^{10}). As d_3^{10} increases, the EM interactions

weaken, and HPP branches become less split (Supplementary Fig. 7c-f). In addition, as momentum k increases, the HPPs are more confined inside the top and bottom ^{11}B slab. EM interactions weaken. The first and second HPP branches become less split and tend to merge at large k. Compared with the first two branches, the high-order HPPs branches are less split since they are more confined inside the ^{11}B slabs.

Supplementary Note 5.2. Engineering HPP dispersions in thickness-asymmetric ^{11}B|^{10}B|^{11}B isotopic heterostructures by varying the ^{10}B slab thickness

In thickness-asymmetric ^{11}B|^{10}B|^{11}B isotopic heterostructures (e.g., top and bottom ^{11}B slab thickness: 40 and 20 nm). Without EM interactions, HPP dispersions in the top and bottom ^{11}B slab follow the red and black curves in Supplementary Fig. 8, respectively. The EM interactions lead to the repulsed[6, 7] HPPs in the ^{11}B|^{10}B|^{11}B heterostructures: the red curve is pushed up, and the black curve is pushed down (Supplementary Fig. 8d-g, false color). As the thickness (d_3^{10}) of the middle ^{10}B slab or k of HPPs increases, the EM interactions weaken, and the two HPP branches gradually approach the original red and black curves. Since the thickness distribution is asymmetric, the dispersions of HPPs in the top and bottom ^{11}B slabs differ. The first and second (from the left) HPP branches in ^{11}B|^{10}B|^{11}B isotopic heterostructures will never merge, in contrast to the case in Supplementary Note 5.1. Similar analysis can be performed for the reciprocal isotopic heterostructures where the thicknesses of the top and bottom ^{11}B slab are 20 and 40 nm, respectively (Supplementary Fig. 8h-k).

In addition, Supplementary Fig. 8reveals branch termination phenomena: the second (from left) HPP branches (initially contributed by the bottom ^{11}B slab) terminate at large k and do not extend to ω ~ 1395 cm^{-1}, in contrast to the long first branches. These branch termination phenomena are attributed to the inadequate large k penetrating down to the bottom ^{11}B slab. Specifically, through the middle ^{10}B slab, all k of the EM fields exponentially decays, and the larger k components decay more significantly. As a result, there are not adequate large k EM fields reaching the bottom ^{11}B slab to excite polaritons. Therefore, the dispersion branches terminate. Note that as the thickness of the middle ^{10}B slab increases, the second HPP branch weakens and terminates at smaller k, in accord with the above-mentioned mechanism. Similarly, one could observe the first (from left) HPP branch termination in the reciprocal isotopic heterostructures (Supplementary Fig. 8h-k).

Supplementary References

1. Dai, S. et al. Efficiency of Launching Highly Confined Polaritons by Infrared Light Incident on a Hyperbolic Material. *Nano Letters* **17**, 5285-5290 (2017).
2. Dai, S. et al. Tunable Phonon Polaritons in Atomically Thin van der Waals Crystals of Boron Nitride. *Science* **343**, 1125-1129 (2014).
3. Giles, A.J. et al. Ultralow-loss polaritons in isotopically pure boron nitride. *Nature Materials* **17**, 134 (2017).
4. Kong, J.A. Electromagnetic Wave Theory. (Wiley, 1986).
5. Chew, W.C. Waves and Fields in Inhomogeneous Media. (IEEE Press, 1995).
6. Fei, Z. et al. Infrared Nanoscopy of Dirac Plasmons at the Graphene–SiO2 Interface. *Nano Letters* **11**, 4701-4705 (2011).
7. Dai, S. et al. Graphene on hexagonal boron nitride as a tunable hyperbolic metamaterial. *Nature Nanotechnology* **10**, 682 (2015).
